# The Dimension of Hyoid Bone Is Independently Associated with the Severity of Obstructive Sleep Apnea

**DOI:** 10.1371/journal.pone.0081590

**Published:** 2013-12-02

**Authors:** Jong Gyun Ha, Hyun Jin Min, Sang Hyeon Ahn, Chang-Hoon Kim, Joo-Heon Yoon, Jeung-Gweon Lee, Hyung-Ju Cho

**Affiliations:** 1 Department of Otorhinolaryngology, Severance Hospital, Yonsei University College of Medicine, Seoul, Korea; 2 Airway Mucus Istitute, Yonsei University College of Medicine, Seoul, Korea; University of Jaén, Spain

## Abstract

**Introduction:**

We hypothesized that the size of the hyoid bone itself may affect the severity of sleep apnea. The aim of this study was to identify the relationship between hyoid bone dimensions and the severity of sleep apnea using computerized tomography (CT) axial images.

**Methods:**

We retrospectively measured the hyoid bone in axial images of neck CTs and correlated these measurements with results of polysomnography in a total of 106 male patients. The new hyoid bone parameters studied in this study were as follows: distance between bilateral lesser horns (LH-d), distance between bilateral greater horns (GH-d), distance from the most anterior end of the hyoid arch to GH-d (AP), distance from the greater to the lesser horn on right and left sides (GH-LH), and the anterior angle between bilateral extensive lines from the greater to the lesser horn (H-angle). Data was analyzed using univariate and multivariate logistic regression, and Pearson correlation tests.

**Results:**

We found a significant inverse correlation between the apnea-hypopnea index (AHI) and GH-d or AP. Neither the LH-d, GH-LH, nor H-angle were associated with the AHI. The patient group that met the criteria of both GH-d<45.4 and AP<33.4 demonstrated the most severe AHI.

**Conclusion:**

The lateral width or antero-posterior length of hyoid bone was associated with AHI and predicted the severity of sleep apnea in male patients. This finding supports the role of expansion hyoidplasty for treatment of sleep apnea. Pre-operative consideration of these parameters may improve surgical outcomes in male patients with sleep apnea.

## Introduction

Obstructive sleep apnea (OSA) is a common disorder that is present in about 4% of men and 2% of women [1]. OSA is characterized by recurrent episodes of upper airway obstruction that occur during sleep and are frequently associated with a reduction in blood oxygen saturation [2]. Repetitive apnea or hypopnea events disturb sound sleep and may lead to systemic hypertension or cardiovascular diseases [3]–[5]. Upper airway obstruction in OSA patients often includes more than one region of the airway. The significance of the structure of the upper airway, including the soft palate, uvula, tongue, and craniofacial skeleton, is well recognized. Therefore it is essential to identify each of these sites using various radiographic images, fiberoptic nasopharyngoscopy, and sleep endoscopy in order to identify major sites of obstruction or to predict the severity of OSA. Among the various methods available for evaluation of OSA, lateral cephalometry is widely used because it is a simple, readily-available, and reproducible method for analyzing craniofacial structures and for diagnosing upper airway obstruction [6], [7]. Cephalometry with other clinical values is very predictable and spares some patients from having to undergo polysomnography. Among the many consistent parameters from lateral cephalometry, the linear distance between the hyoid bone and the mandibular plane (H-MP) is commonly used. Its longer distance has been shown to correlate with OSA [6]. However, the limitation of lateral cephalometry is that it only can show the antero-posterior dimension, but not lateral width. Drug-induced sleep endoscopy (DISE) is often used to identify the site of airway obstruction, but there is no association between lateral cephalometry and DISE findings [8]. Conventional uvulopalatopharyngoplasty primarily addresses the antero-posterior dimension. However, lack of the lateral dimension and the collapsibility of the lateral pharyngeal wall are factors which may be important causes of the high failure rate of conventional uvulopalatopharyngoplasty [9].

**Figure 1:**
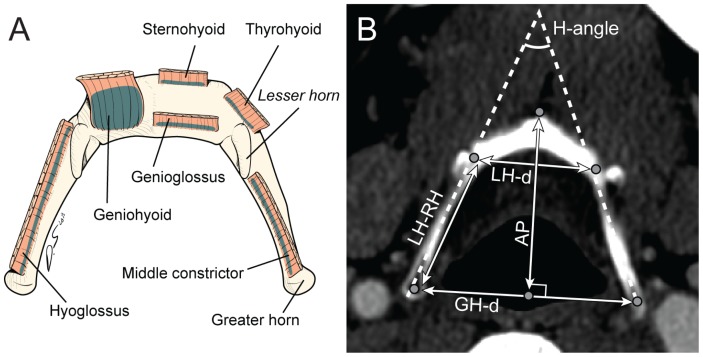
Anatomy of hyoid bone and new parameters. **A**. Schematic of the hyoid bone and attachment sites with the upper airway pharyngeal muscles. **B**. Axial image of the hyoid bone on a computed tomographic (CT) scan. Parameters that were measured are indicated on the CT image and include the following: *LH-d*, distance between the bilateral lesser horns; *GH-d*, distance between bilateral greater horns; *AP*, distance from the most anterior end of hyoid arch to GH-d; *H-angle*, angle made by bilateral extension lines between greater and lesser horn; *GH-LH*, distance between greater and lesser horns.

The hyoid bone does not articulate with any other bony structure. But, it is an important anatomical structure that plays an important role in OSA. Muscles attached on the hyoid bone include the geniohyoid, genioglossus, sternohyoid, and thyrohyoid (Figure 1A). The position of the hyoid is determined by the coordination of these muscles. Another muscle inserted on the hyoid is middle pharyngeal constrictor muscle that forms the pharyngeal wall and affects the luminal airway volume or its collapsibility.

Therefore, we hypothesized that the dimensions of the hyoid bone in OSA patients might be different compared to normal subjects. And, patients with severe OSA may have a small hyoid bone. Lateral cephalometry is a useful method for determining size, but unable to show the size of the hyoid bone in various dimensions. Thus, we utilized axial images from CT to measure length in various dimensions.

## Materials and Methods

One hundred and six subjects were analyzed. Patients ranged in age from 19 to 77 years old (mean age; 45.6±13.0 y old). The present study only included male patients, because the features of OSA in men and women differ. Furthermore, upper airway size correlates significantly with the severity of sleep apnea only in men [10]. Sexual dimorphism of the adult hyoid bone was another reason that we included only male patients in our study [11]. We retrospectively reviewed the medical records of patients who visited the sleep clinic in the Department of Otorhinolaryngology at Yonsei University Severance Hospital. Data from patients who received overnight polysomnography and paranasal sinus CT were analyzed. These data included imaging of the hyoid bone. The apnea-hypopnea index (AHI) was calculated as the sum of the total events of the apnea index and hypopnea index. Apnea was defined as the absence of airflow for 10 seconds or longer. Hypopnea was defined as reduced airflow in at least 50% of the thoraco-abdominal belts (also compared to prior epochs) with the presence of either oxygen desaturation ≥4% of the normal level or an arousal.

Axial images of the hyoid bone were obtained by a paranasal sinus CT, which allowed analysis of the nose and upper airway structure. All measurements were accomplished with using image software (Centricity Radiology RA1000, General Electric Company, USA). The following five parameters were evaluated as shown in Figure 1B: distance between bilateral lesser horns (LH-d), distance between bilateral greater horns (GH-d), distance from the most anterior end of the hyoid arch to GH-d (AP), distance from the greater to lesser horn (GH-LH), and the anterior angle between bilateral extensive lines from the greater to lesser horn (H-angle).

This study was approved by the Institutional Review Board of Yonsei University College of Medicine (4-2013–0478). The written consent was also given by the patients for their information to be stored in the hospital database and used for research.

### Statistical analysis

The odds ratios (ORs) of variable factors were determined by univariate and multivariate logistic regression analyses. Factors with a P value<0.05 in the univariate analysis were further examined by multivariate logistic regression analyses. Adjusted odds ratios with 95% confidence intervals were calculated for these independent variables. Pearson correlation coefficients were analyzed between variables and polysomnographic findings. Statistical analyses were performed with SPSS version 2.0 (IBM Corporation, Armonk, NY, USA).

## Results

**Figure 2:**
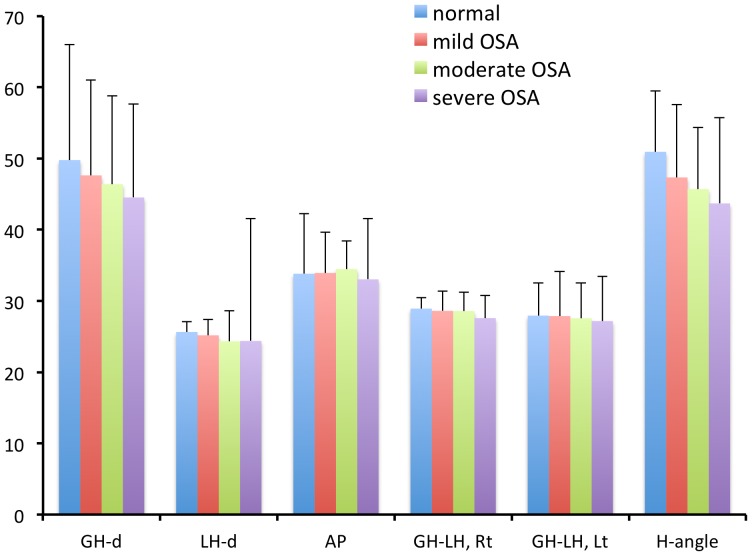
Comparison of hyoid bone parameter values in four groups of patients with sleep apnea.

One hundred and six male patients with normal to severe OSA were analyzed in this study. The mean age was 45.6±13.0 years (y). Patients were divided into four groups according to the AHI. The numbers of subjects, age, and AHI from each group were as follow: normal (n = 5, 43.8±16.2 y, AHI = 2.8±1.4), mild (n = 18, 46.5±13.4 y, AHI = 10.6±2.2), moderate (n = 18, 44.5±12.4 y, AHI = 21.0±12.4), and severe (n = 65, 45.6±13.1 y, AHI = 56.2±17.2). Physical, polysomnographic, and other parameters measured from the hyoid bone are summarized in Table 1. A shorter or narrower GH-d or H-angle indicated a tendency toward more severe OSA (Figure 2).

**Table 1:** Patients characteristics.

	normal	mild OSA	moderate OSA	severe OSA
age (y)	43.8±16.2	46.5±13.4	44.5±12.4	45.6±13.1
AHI	2.8±1.4	10.6±2.2	21.0±4.3	56.2±17.2
Lowest O_2_ sat (%)	86.5±8.5	85.5±5.8	84.2±3.9	76.2±8.5
BMI	24.0±1.5	24.3±2.8	24.6±2.6	27.2±3.2
Height (cm)	173.2±4.5	172.4±6.3	174.2±5.0	171.3±6.3
Weight (kg)	72.6±8.5	71.8±10.2	74.7±8.6	80.0±12.0
Neck circumference (cm)	37.9±1.4	38.6±2.9	38.4±2.2	40.0±2.4
Waist circumference (cm)	85.9±3.7	88.4±6.6	88.1±7.3	94.2±9.6
Head circumference (cm)	94.6±3.9	95.6±4.0	95.8±5.4	99.3±6.0
ESS	12.2±5.8	10.7±4.7	9.2±4.8	11.6±4.4
GH-d (mm)	49.7±8.6	47.6±5.1	46.4±7.1	44.5±5.6
LH-d (mm)	25.6±0.7	25.2±2.6	24.3±2.6	24.4±2.5
AP (mm)	33.8±4.2	33.9±2.8	34.5±4.7	33.0±3.7
GH-LH, Rt (mm)	28.9±3.1	28.6±2.5	28.6±3.3	27.6±3.3
GH-LH, Lt (mm)	27.9±3.7	27.9±2.2	27.6±3.8	27.2±3.1
H-angle (°)	50.9±17.0	47.3±11.3	45.7±15.9	43.7±12.5
Total number	5	18	18	65

Data are presented as mean±SD for data with normal distribution.

AHI: apnea/hypopnea index, BMI: body mass index, ESS: Epworth sleepiness score.

GH-d: distance between bilateral greater horns, LH-d: distance between bilateral lesser horns, AP: distance from most anterior end of hyoid arch to GH-d, GH-LH, Rt: distance from the right greater to right lesser horn, GH-LH, Lt: distance from the left greater to left lesser horn, H-angle: distance from greater to lesser horn, and anterior angle between bilateral extensive lines from greater to lesser horn.

**Figure 3:**
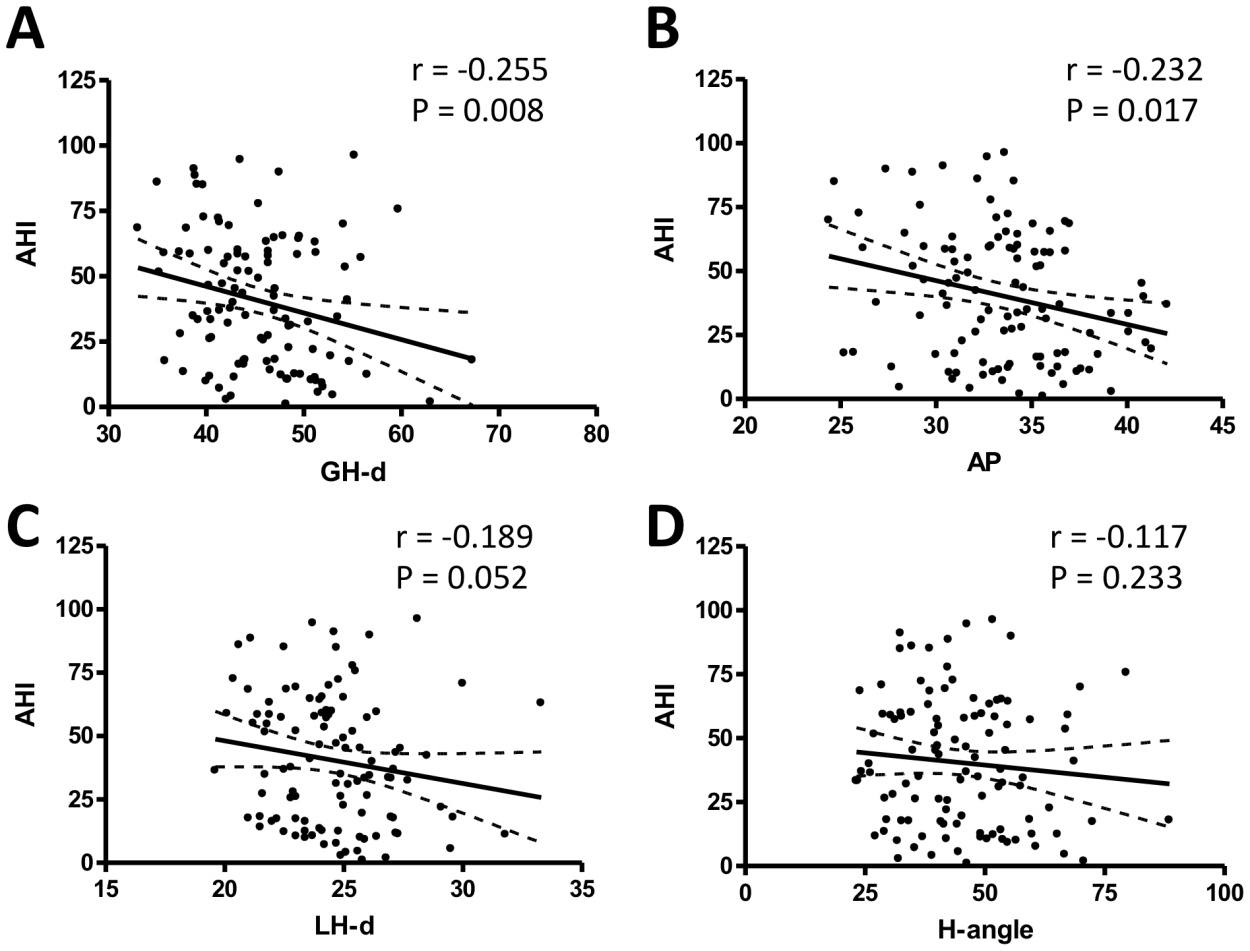
Correlation between apnea-hypopnea index (AHI) and parameters of the hyoid bone. **A**. GH-d is inversely correlated to the AHI (r = −0.255, P = 0.008). **B**. AP is inversely correlated to the AHI (r = −0.232, P = 0.017). C. LH-d has no correlation with the AHI (r = −0.189, P = 0.052). D. H-angle has no correlation with the AHI (r = −0.117, P = 0.233).

Typically, the AHI was examined as a diagnostic for OSA. Correlations between the AHI and five parameters related to the hyoid bone were tested further (Table 2). The GH-d showed a significant inverse correlation with the AHI (r = −0.255, P = 0.008, Figure 3A). A shorter GH-d was associated with a higher AHI. The AP was another noteworthy parameter that showed a significant inverse correlation with the AHI (r = −0.232, P = 0.017, Figure 3B). Patients with shorter AP values had higher AHI values. However, the LH-d did not correlate with the severity of the AHI (r = −0.189, P = 0.052, Figure 3C). The distance between the greater and lesser horn (GH-LH) or H-angle did not correlate with the AHI. In contrast to our expectations, the H-angle also did not correlate with the AHI (r = −0.117, P = 0.233, Figure 3D). The lowest O_2_ saturation was analyzed in the same manner as the AHI. In contrast to the AHI, the lowest O_2_ saturation did not significantly correlate with the hyoid bone parameters (Table 2).

**Table 2:** Correlation of AHI and lowest O2 saturation with hyoid bone parameters.

	Parameters	r	p
	LH-d	−0.189	0.052
	GH-d	−0.255	0.008 **
AHI	AP	−0.232	0.017 *
	GH-LH-Rt	−0.130	0.184
	GH-LH-Lt	−0.172	0.078
	H-angle	−0.117	0.233
	LH-d	0.027	0.781
	GH-d	0.041	0.673
Lowest O_2_	AP	0.134	0.172
saturation	GH-LH-Rt	0.107	0.274
	GH-LH-Lt	0.085	0.388
	H-angle	−0.001	0.996

AHI: apnea/hypopnea index.

r: Pearson correlation; *: significant (p<0. 05), **: significant (p<0. 01).

LH-d: distance between bilateral lesser horns, GH-d: distance between bilateral greater horns, AP: distance from most anterior end of hyoid arch to GH-d, GH-LH, Rt: distance from the right greater to right lesser horn, GH-LH, Lt: distance from the left greater to left lesser horn, H-angle: distance from greater to lesser horn, and anterior angle between bilateral extensive lines from greater to lesser horn.

The LH-d, GH-d, and AP were selected and further investigated by univariate and multivariate logistic regression analyses (Table 3). The GH-d and AP were significantly identified by multivariate analyses (P = 0.005 and P = 0.004). The odds ratio for the GH-d and AP were −1.234 and −1.879, respectively. These data indicated that a shorter GH-d or AP increased the probability of a severe AHI. However, the LH-d was not related to the AHI according to multivariate analysis (P = 0.909).

**Table 3:** Odds ratio of LH-d, GH-d, and AP for AHI by univariate and multivariate analysis.

	Parameters	Univariate OR OR (95% CI)	P value	Multivariate OR OR (95% CI)	P value
	LH-d	−1.925	0.052	−0.122	0.909
AHI	GH-d	−1.045	0.008	−1.234	0.005 **
	AP	−1.535	0.017	−1.879	0.004 **

** : significant (p<0. 01).

OR =  odds ratio, CI =  confidence interval.

LH-d: distance between bilateral lesser horns, GH-d: distance between bilateral greater horns, AP: distance from most anterior end of hyoid arch to GH-d.

**Figure 4:**
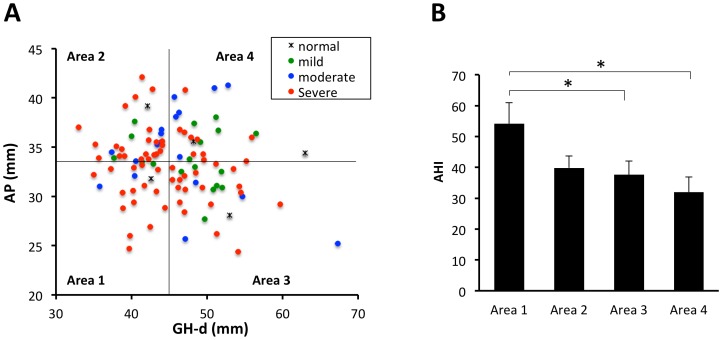
Comparison of four groups of patients with sleep apnea divided by mean length of GH-d and AP. **A**. Plot of all subjects (n = 101) by GH-d and AP. All plots were divided into four areas according to the mean values of GH-d and AP. The mean value of GH-d was 45.4±5.9 mm, and AP was 33.4±3.8 mm. A*rea 1*, GH-d<45.4 and AP<33.4; *area 2*, GH-d<45.4 and AP≥33.4; *area 3*, GH-d≥45.4 and AP<33.4; *area 4*, GH-d≥45.4 and AP≥33.4. **B**. The mean AHI from four areas were compared. The mean AHI of *area 1* was 54.2±26.2, which was significantly higher than *area 3* (37.6±23.3) or *area 4* (31.9±24.9). (t-test, p = 0.04 and 0.01, respectively).

We plotted all of the subjects by their GH-d and AP values in an X-Y plot (Figure 4A). The mean GH-d from all of the OSA patients (n = 101) was 45.4±5.9 mm, and the mean AP was 33.4±3.8 mm. Each plot was divided into four areas according to the mean GH-d and AP values: *area 1*, GH-d<45.4 and AP<33.4; *area 2*, GH-d<45.4 and AP≥33.4; *area 3*, GH-d≥45.4 and AP<33.4; *area 4*, GH-d≥45.4 and AP≥33.4. The mean AHI for each of the four areas was obtained and compared (Figure 4B). The mean AHI of *area 1* (54.2±26.2) was significantly higher than that of *area 3* (37.6±23.3) or *area 4* (31.9±24.9) by t-test (P = 0.04 and 0.01, respectively).

## Discussion

The severity of sleep apnea increases if obstructions exist at both the retropalatal and retroglossal regions. Identification of the obstructed sites is crucial for obtaining a high success rate from surgery. Recently, numerous imaging techniques, including lateral cephalometry, computed tomography, MRI, and drug-induced sleep endoscopy have been used to improve knowledge regarding the relationship between the upper airway structures and OSA [12]. Published analyses regarding factors related to the hyoid bone are limited. The distance between the mandibular plane and the hyoid bone, which can be determined by lateral cephalometry, is the only parameter that has been reported to be associated with the severity of sleep apnea [13]. The hyoid bone is usually located at a lower and more posterior position in a patient with OSA compared to a control subject [14]. We supposed that hyoid bone might play a more significant anatomical role than previously realized because the hyoid bone serves as a platform for various pharyngeal muscles and contributes to the structure of the pharyngeal lumen.

This is the first study to report new parameters related to the hyoid bone as indicators of obstructive sleep apnea severity. Our initial idea for this study came from an earlier study that tested expansion hyoidplasty in a canine model [15]. Expansion hyoidplasty involves transecting just medial to the lesser horn, moving each greater horn laterally, and suspending the hyoid body toward the inner surface of the mandible to shift the base of the tongue in an anterior direction [15]. Increased airflow and reduced closing pressure of the upper airway were achieved with expansion hyoidplasty in the canine model. Human cadaveric study also showed the increase of retrolingual hypopharyngeal airway space with hyoid expansion [16]. The greater horn is a significant structure. The middle constrictor and hyoglossus are attached. Lateral movement of these muscles might allow lateral expansion of the hypopharyngeal wall, which would improve sleep apnea. Our result that GH-d was inversely related with the severity of OSA supports this concept. The hyoid body is a structure where the geniohyoid and genioglossus muscle attach, and anterior movement widens the antero-posterior dimension of the retroglossal area. Based on this study, Hamans et al. tried a modified hyoid expansion technique for patients with sleep apnea in a prospective multicenter pilot study [17]. They bisected the hyoid bone via a midline osteotomy and applied an implantable device (the Air-Frame system) to cause a 10-mm-lateral expansion of the hyoid bone. The feasibility, safety, and post-operative morbidity were evaluated and found to be reasonable. The efficacy was assessed by the AHI, the Snore Outcomes Survey (SOS) to monitor snoring, and the Epworth Sleepiness Scale (ESS) to monitor daytime sleepiness. The procedure improved snoring and daytime sleepiness significantly but failed to show consistent improvement of the AHI. The modified hyoid expansion technique expands only the lateral dimension of the hyoid bone and may have been insufficient for increasing the airway lumen. In addition, the shape and size of the hyoid bone were not considered. Thus, the effect of the surgery might have been minimal in the preoperative hyoid bone if there the length and width were sufficient. Another potential explanation is that the surgical method was not clearly described. A multicenter study involves surgeons from different centers with potentially different surgical approaches, which may cause a bias to the surgical outcome.

There are some procedures that are related to the hyoid bone, including the modified hyoid suspension technique. This technique anchors the hyoid bone to thyroid cartilage and is a framework surgery for hypopharyngeal obstruction in sleep apnea [18]. This technique significantly improves the excessive daytime sleepiness and polysomnographic features present in patients with OSA [18], [19]. However, MRI studies performed before and after surgery show that the clinical effect of hyoid suspension is more likely due to prevention of functional airway collapse rather than direct enlargement of airway space [20]. Therefore, modified hyoid suspension alone is not an effective treatment. Combination surgery with genioglossus advancement or tongue radiofrequency ablation are also recommended [21].

Our results did not show that widening the hyoid bone increases the airway lumen or causes outstanding success. However, we believe that tension around the airway musculature should be elevated according to a previous study [15]. This might help prevent airway collapse in certain patients with small hyoid bones. The small hyoid bone to which upper airway muscles attach is very crowded. Thus, the pharyngeal wall may become weaker, causing collapse. The lateral lengthening of the bilateral greater horns will increase the tension or lateral dimension of the pharynx by expanding the middle constrictor muscle and the neighboring superior constrictor muscle. Expansion of the antero-posterior dimension of the hyoid bone will also enhance those effects.

Lateral wall collapse of the pharyngeal segment participates in the pathogenesis of OSA. The role of the lateral wall has been observed by CPAP during drug-induced sleep endoscopy [22] or MRI [9]. Various modifications of uvulopalatopharyngoplasty (UPPP), such as lateral pharyngoplasty [23] or expansion sphincter pharyngoplasty [24], have been developed to repair the lateral pharyngeal wall by surgery. These procedures effectively enhance lateral wall tension by reconstructing musculature at the retropalatal level and have shown better results than conventional UPPP. We expect that a combination of surgical techniques that enlarge the hyoid bone area with effective palatal surgery will be very effective for improving the surgical outcomes for patients with sleep apnea.

This study has some limitations. First, we had a small number of subjects whose AHI was less than 5 by polysomnography. Our hospital is a tertiary medical center, and most patients were referred for evaluation of severe snoring or sleep apnea. Second, subjects with various sizes of tonsils were included in the analysis. Tonsil size can affect the severity of sleep apnea. Although the relationship between tonsil grade and parameters of the hyoid bone were not investigated in this study, we hypothesize that the presence of a large tonsil since childhood can intensify negative pressure in the lumen of the upper airway, which restricts growth of the hyoid bone. We found that the size of the hyoid bone was not associated with height. Thus, values were not normalized by height. Instead, the GH-d negatively correlated with BMI or body weight in our analysis (data not shwon). Third, only male patients were included in this study. Females have not yet been analyzed due to a small number of subjects. However, we will perform this analysis in the future. Finally, the shape of airway collapse during sleep has not yet been investigated but can be done by DISE. Thus, additional studies with DISE regarding the association of patterns of airway collapse and hyoid parameters are being performed. These studies may support a new mechanism for upper airway dynamics in patients with sleep apnea.

## Conclusion

In this study, new hyoid parameters such as GH-d or AP were statistically associated with AHI in OSA male patients. This finding supports the role of expansion hyoidplasty for treatment of sleep apnea. These hyoid parameters should be carefully reviewed before surgical treatment and it would improve surgical outcomes in male patients with sleep apnea.
